# A bird’s eye view of mitochondrial unfolded protein response in cancer: mechanisms, progression and further applications

**DOI:** 10.1038/s41419-024-07049-y

**Published:** 2024-09-11

**Authors:** Xinyu Zhang, Yumei Fan, Ke Tan

**Affiliations:** Ministry of Education Key Laboratory of Molecular and Cellular Biology; Hebei Research Center of the Basic Discipline of Cell Biology, Hebei Collaborative Innovation Center for Eco-Environment, Hebei Province Key Laboratory of Animal Physiology, Biochemistry and Molecular Biology, College of Life Sciences, Hebei Normal University, Shijiazhuang, Hebei China

**Keywords:** Mitochondria, Cancer

## Abstract

Mitochondria are essential organelles that play critical roles in energy metabolism, apoptosis and various cellular processes. Accumulating evidence suggests that mitochondria are also involved in cancer development and progression. The mitochondrial unfolded protein response (UPR^mt^) is a complex cellular process that is activated when the protein-folding capacity of the mitochondria is overwhelmed. The core machinery of UPR^mt^ includes upstream regulatory factors, mitochondrial chaperones and proteases. These components work together to eliminate misfolded proteins, increase protein-folding capacity, and restore mitochondrial function. Recent studies have shown that UPR^mt^ is dysregulated in various cancers and contributes to tumor initiation, growth, metastasis, and therapeutic resistance. Considering the pivotal role of the UPR^mt^ in oncogenesis, numerous compounds and synthetic drugs targeting UPR^mt^-related components induce cancer cell death and suppress tumor growth. In this review, we comprehensively summarize recent studies on the molecular mechanisms of UPR^mt^ activation in *C. elegans* and mammals and elucidate the conceptual framework, functional aspects, and implications of the UPR^mt^ for cancer therapy. In summary, we paint a developmental landscape of the UPR^mt^ in different types of cancer and offer valuable insights for the development of novel cancer treatment strategies by targeting the UPR^mt^.

## Facts

- Various molecules and multiple mechanisms participate in the UPR^mt^ activation in *C. elegans* and mammals.
- Activation of UPR^mt^ promotes the invasion and metastasis of cancer cells.
- The fluctuation of UPR^mt^ is associated with a variety of physiological processes and diseases.
- UPR^mt^ manipulation is a potential therapeutic target for treating human cancers.

## Open Questions

- How do seemingly independent key components interact with each other in the molecular mechanism of UPR^mt^ activation?
- Do different signaling pathways of UPR^mt^ jointly regulate the occurrence and development of tumors?
- What is the clinical efficacy of potential anti-cancer drugs targeting UPR^mt^?
- By what mechanism does overactivation of UPR^mt^ negatively affect cancer cells, and can we use this process to treat tumors?

## Introduction

Mitochondria, referred to as the “powerhouses of the cell”, have a multitude of functions that are essential to cell survival and organismal health [1, 2]. Mitochondria not only produce the majority of the cell’s ATP through oxidative phosphorylation (OXPHOS) but also regulate calcium levels and apoptosis within the cell [3, 4]. Mitochondria are also the hub of various metabolic pathways, including the TCA cycle, fatty acid oxidation and amino acid metabolism [5, 6]. Considering the critical roles of mitochondria, the number and function of mitochondria are strictly regulated to adapt to various environmental stimuli.

As a complex and finely regulated organelle, the proper functioning of mitochondria often faces internal or external pressures. Fortunately, cells have the ability to cope with the stress conditions they encounter. Due to the presence of multiple compartments in cells, different and intricate pathways have evolved in the cytoplasm, endoplasmic reticulum (ER) and mitochondria to ensure dynamic balance of proteins, known as heat shock response (HSR) and ER stress (UPR^ER^) and mitochondrial unfolded protein reactions (UPR^mt^), respectively. They are precisely coordinated, not only requiring close communication with the nucleus, but also influencing each other [7]. For example, classical ER stress inducers can modulate the expression of three core transcriptional regulators, including activating transcription factor 4 (ATF4), ATF5 and CCAAT/enhancer-binding protein homology protein (CHOP), which are required for UPR^mt^ induction [8, 9] (for crosstalk between UPR^ER^ and UPR^mt^, see Supplementary file S1). Due to the fact that mitochondria are a major source of reactive oxygen species (ROS), ROS have also become an important medium for communication between mitochondrial stress and other cellular stresses (for ROS and UPR^mt^, see Box 1). More importantly, the crosstalk between mitochondria and other organelles allows for the integration of UPR^mt^ with broader cellular stress responses, ensuring the maintenance of cellular homeostasis and the resolution of stress.

The association between the UPR^mt^ and cancer development has been gradually explored. Mitochondria play important roles in all stages of cancer cell development [10]. During tumor growth, mitochondria exposed to stress also facilitate cancer development because mitochondria have unique mechanisms to maintain their stability, including the UPR^mt^. Activation of the UPR^mt^ improves the invasion and metastasis of cancer cells [11]. Cancer cells slyly utilize this self-repair mechanism of mitochondria to accelerate their proliferation. Thus, in cancer cells, the UPR^mt^ is hijacked and exploited for the repair of mitochondria and the promotion of oncogenesis. Disrupting the proteostasis in cancer cells by targeting UPR^mt^ is considered a novel anticancer therapeutic strategy.

Box 1 Mitochondrial ROS and UPR^mt^ in cancer: A balancing act Oxidative stress leads to protein misfolding in mitochondria, triggering the UPR^mt^. ROS are generated by mitochondria as a by-product of the electron transport chain during OXPHOS. Mitochondria have their own DNA (mtDNA) and are more susceptible to oxidative damage. Excessive ROS directly damage mitochondrial proteins, lipids and mtDNA. Mitochondrial protein dysfunction, including oxidation and denaturation, significantly activates the UPR^mt^ to restore proteostasis by the guidance of mitochondrial chaperonin and proteases. If the UPR^mt^ fails to effectively resolve the stress, such as in cases of severe or prolonged oxidative stress, mitochondrial function continues to decline and ROS are further produced, creating a positive feedback loop. Notably, ROS also play a multifaceted role in oncogenesis, acting as a double-edged sword. At physiological levels, ROS serve as crucial signaling molecules in a multitude of cellular functions, including cancer cell growth, proliferation and differentiation. Once they exceed the normal physiological range, ROS induce cellular damage and cancer cell death.

### Overview of UPR^mt^

The UPR^mt^ is a relatively independent life process that was first discovered in mammalian cells. After arduous efforts, the UPR^mt^ is analyzed in detail in nematodes. The UPR^mt^ is relatively conserved between nematodes and mammals, but more complex regulatory mechanisms in mammals have been gradually discovered [12, 13]. The UPR^mt^ of mammals is regulated by different signaling pathways and has multiple executors. The existence of multiple pathways provides a level of redundancy to the system. If one pathway or regulatory factor becomes dysfunctional, other pathways can compensate to maintain the cell’s ability to respond to mitochondrial stress and ensure cell survival.

### The molecular mechanism of the UPR^mt^ in *C. elegans*

#### ATFS-1 and UPR^mt^

**Fig. 1:**
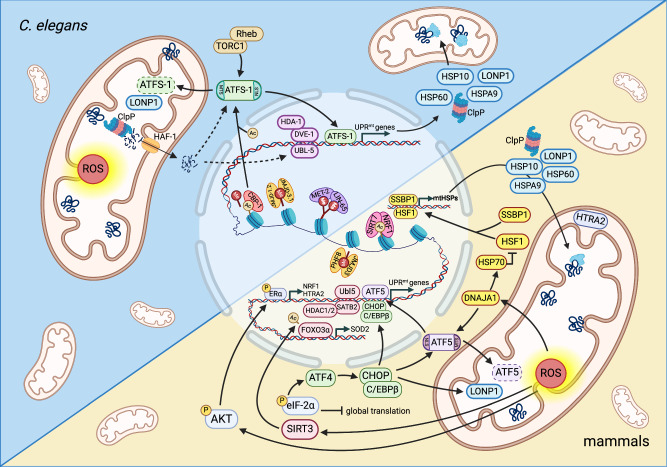
Summary of the molecular mechanisms of UPR^mt^ in *C. elegans* and mammals. In *C. elegans*, the misfolded proteins in mitochondria are digested by ClpP and transported to the cytoplasm by HAF-1. The reduced efficiency of protein input is an indicator of mitochondrial dysfunction and triggers UPR^mt^. During UPR^mt^, ATFS-1 moves to the nucleus to promote the expression of many UPR^mt^-related genes, resulting in increased synthesis of mitochondrial proteins, such as HSP10 and LONP1, to help cells survive. The chromatin remodeling factors UBL-5 and DVE-1 form complexes that interact with promoters of UPR^mt^-related genes, which may help ATFS-1 bind to nuclear DNA. Additionally, several chromatin remodeling factors function dependently of ATFS-1. In mammals, ATF5 plays a similar role to ATFS-1. Phosphorylated eIF2α upregulates the expression of ATF4 and CHOP, and then CHOP dimerizes with C/EBPβ. The expression of ATF5 is affected by ATF4 and CHOP. Independently, AKT phosphorylates and activates ERα to modulate NRF1 and HTRA2 expression. HSF1 binds with SSBP1 and modifies chromosomes to increase the transcription of HSP10, HSP60 and HSPA9 to stimulate UPR^mt^. Moreover, PHF8 and JMJD3 affect the expression of UPR^mt^-related genes through a conserved epigenetic mechanism. Figure was generated utilizing Biorender.com.

Accumulating evidence suggests that the bZIP transcription factor ATFS-1 (activating transcription factor associated with stress-1) plays a pivotal role in the UPR^mt^ [13, 14]. It is activated in response to mitochondrial stress and functions as a central transcriptional regulator of the UPR^mt^ in *C. elegans*. ATFS-1 contains a nuclear localization sequence (NLS) and a mitochondrial targeting sequence (MTS) [12], making ATFS-1 a medium for mitochondria to transmit signals to the nucleus [12, 14]. Under normal conditions, ATFS-1 enters mitochondria to facilitate its degradation. Following mitochondrial stress, ATFS-1 moves more to the nucleus, possibly due to a reduction in mitochondrial membrane potential or a change in the efficiency of transporting proteins into mitochondria [14–16]. Then ATFS-1 promotes the expression of UPR^mt^-related genes by directly binding to the promoters, thereby supporting cells to overcome mitochondrial dysfunction (Fig. 1). A recent interesting study proposes that changes in tRNA processing in *C. elegans* could activate the UPR^mt^ [17]. The upregulation of nuclear HOE-1 increases 3’-tRNA processing, resulting in the accumulation of nuclear ATFS-1 and DVE-1.

In the process of ATFS-1-activated UPR^mt^, mitochondrial misfolded proteins are digested into small peptides by the mitochondrial matrix-localized caseinolytic protease P (ClpP), and transported to the cytoplasm by inner mitochondrial membrane-located HAF-1 (Fig. 1) [18]. Both ClpP and HAF-1 participate in UPR^mt^ activation. The contribution of HAF-1 in peptide efflux prevents ATFS-1 from entering the mitochondria [14]. A recent study demonstrates that mitochondria activate the target of rapamycin complex 1 (TORC1) through v-ATPase- and Rheb-dependent mechanisms following stress, resulting in increased translation of ATFS-1 to ensure that enough ATFS-1 translocate to the nucleus to stimulate the UPR^mt^ (Fig. 1) [19]. In fact, the regulatory effect of ATFS-1 on mitochondrial function may far exceed our current understanding. For instance, ATFS-1 promotes mtDNA replication by promoting the binding of mitochondrial DNA replication polymerase (POLG) to mtDNA [20]. Additionally, ATFS-1 interferes with the assembly of mitochondrial preinitiation transcription complexes to inhibit transcription and aid mtDNA repair to ensure mitochondrial function [21]. With the deepening of research, more and more functions of ATFS-1 have been described.

#### Chromatin remodeling factors and UPR^mt^

During the movement of ATFS-1 to the nucleus, ubiquitin-like protein 5 (UBL-5) is upregulated with the assistance of ATFS-1 and defective proventriculus homolog protein (DVE-1) [22]. Subsequently, UBL-5 and DVE-1 form a complex that interacts with the promoters of UPR^mt^-related genes for chromatin remodeling, which may facilitate the binding of ATFS-1 to nuclear DNA (Fig. 1) [12]. The discovery of the homologs of DVE-1 and UBL-5 in mammals (SATB2 and Ubl5) again confirms the conservation of UPR^mt^. Notably, there are many chromatin remodeling factors that play a role in UPR^mt^ activation. For example, the cytosolic protein LIN-65 is an indispensable component for full activation of UPR^mt^ and plays a specific role in mitochondrial stress but not in cytoplasmic or ER stress [23]. LIN-65 moves toward the nucleus under the action of histone methyltransferase MET-2 during mitochondrial stress to dimethylate histone H3K9 to silence most of the chromatin (Fig. 1). Moreover, LIN-65 and MET-2 also affect the expression and distribution of DVE-1 in the nucleus, while DVE-1 influences the expression and distribution of LIN-65, suggesting that there is crosstalk among these factors [23]. Genes are almost turned off by LIN-65 and MET-2, but if transcription of all genes is suppressed, this clearly contradicts with the effect of UPR^mt^. Therefore, the expression of some substances produced in large quantities during the UPR^mt^, such as chaperone HSP-6 and protease YMEL-1, requires special mechanisms for targeted activation. JMJD-1.2 and JMJD-3.1, conserved histone demethylases of the JumonjiC (JmjC)-domain-containing protein family, are involved in longevity and are necessary for UPR^mt^ excitation [24]. They act upstream of ATFS-1 and perform similar but not identical modifications to histones. JMJD-1.2 and JMJD-3.1 regulate the status of UPR^mt^-associated genes through their demethylase activity, allowing these essential genes to be transcribed to relieve stress on mitochondria (Fig. 1).

CREB-binding protein-1 (CBP-1) is the worm ortholog of the mammalian acetyltransferase CBP/p300. CBP-1 enhances the protective response to polystyrene nanoparticles in certain tissues and regulates UPR^mt^ activation in *C. elegans* [25]. Specifically, CBP-1 can convert histone methylation to acetylation to promote the transcription of UPR^mt^-related genes (Fig. 1) [26]. This step occurs downstream of JMLD-1.2 and JMLD-3.1 and upstream of ATFS-1, transmitting mitochondrial emergency signals to the nucleus. In mammals, PHF8 and JMJD3 also play similar roles as homologs of JMJD-1.2 and JMJD-3.1, affecting the expression of UPR^mt^-related genes [27]. CBP-1 also directly acetylates ATFS-1, indicating that CBP-1 may play multiple and reversible roles in the UPR^mt^.

In *C. elegans*, histone deacetylase HDA-1, the homolog of mammalian histone deacetylase (HDAC), cooperates with DVE-1 to activate UPR^mt^ [28]. Consistently, in mammals, HDAC1/2, together with SATB2, maintains mitochondrial homeostasis and assists other chromatin-remodeling enzymes in their function. Overall, when UPR^mt^ is triggered, many epigenetic modification-related factors are systematically regulated to activate UPR^mt^ [29].

In addition to the UPR^mt^ triggered by internal or external stresses on the cell itself, stressed mitochondria also communicate with other mitochondria in distant cells, and signals from the UPR^mt^ can be transmitted between different tissues. The regulation of stress response by this cell non-autonomous mechanism is considered an important way to affect body health [29]. (for further discussion about cell non-autonomous UPR^mt^, see Supplementary file S2).

### The molecular mechanism of the UPR^mt^ in mammals

#### ATF5 and UPR^mt^

Accumulating evidence proves that various stresses lead to ATF5-mediated UPR^mt^ activation, and loss of ATF5 reduces the expression of UPR^mt^-related genes. ATF5 is the homolog of ATFS-1 in mammals. ATF5 plays similar roles as ATFS-1, and ATF5 can compensate for the loss of ATFS-1 under stress [12]. ATF5 also has MTS and NLS; thus, it can translocate into mitochondria and sense their state, thereby transmitting signals to the nucleus. Under normal conditions, ATF5 localizes to mitochondria and maintains the survival state of mitochondria. Knockdown of ATF5 significantly reduces basal, overall and maximal respiration [30]. During mitochondrial stress, ATF5 translocates into the nucleus and forms dimers with other transcription factors to regulate downstream target genes, including mitochondrial chaperones and proteases (Fig. 1) [10, 31]. Thus, ATF5 acts specifically in mitochondria, and ATF5 activity correlates with mitochondrial protein input efficiency [32]. A recent study reveals that tubular interstitial injury is related to the activation of the UPR^mt^, and ATF5 expression in the kidney is positively correlated with the expression of UPR^mt^-related molecules. Knockout of ATF5 alleviates tubular oxidative stress and apoptosis by coordinating the UPR^mt^ pathway [33]. Moreover, inhibition of ATF5 activity leads to inhibition of the UPR^mt^, resulting in defects in monocyte development [34]. Knockdown of ATF5 reduces the production of proinflammatory cytokines and tumor necrosis factor-α (TNF-α) by inhibiting the UPR^mt^; thus, ATF5 may become a therapeutic target for alleviating neuroinflammatory processes [35].

#### ATF4, CHOP and UPR^mt^

**Fig. 2:**
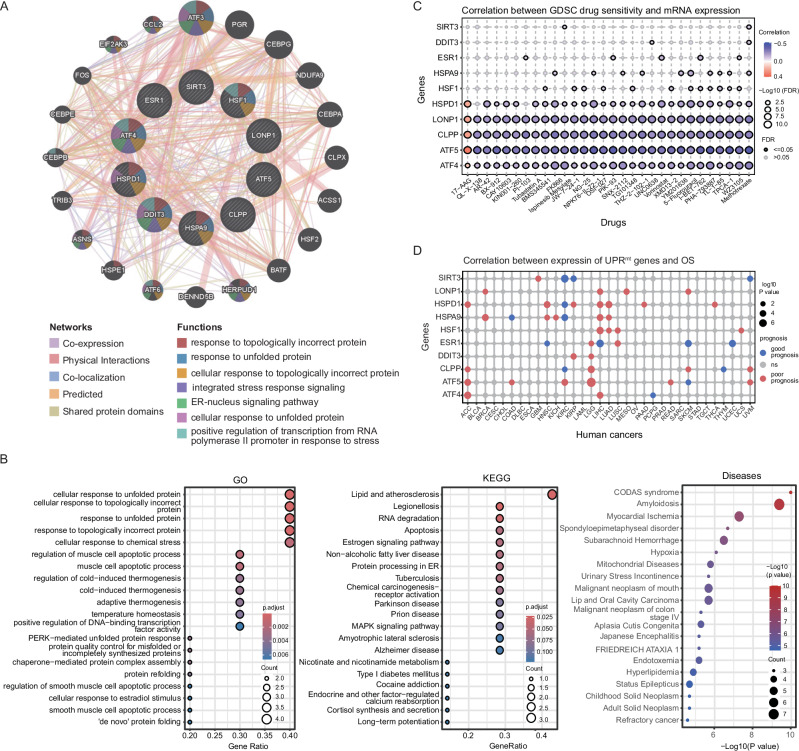
Bioinformatics analysis of 10 core UPR^mt^ genes-related signaling pathways and human diseases, as well as their correlation with clinical drugs. **A** The protein-protein interaction (PPI) network is analyzed using the GeneMANIA database (http://genemania.org/). **B** Gene Ontology (GO) and Kyoto Encyclopedia of Genes and Genomes (KEGG) analyses of 10 core UPR^mt^-related genes were performed using the R package clusterProfiler and visualized using the R package enrichplot. The gene-disease associations in DisGeNET database were explored using the R package disgenet2r. **C** The correlations between gene expression and the sensitivity of drugs (top 30) in pan-cancer were analyzed using The Genomics of Drug Sensitivity in Cancer (GDSC) database. Positive correlation suggests that increased gene expression may contribute to drug resistance, whereas negative correlation implies that elevated gene expression may enhance drug sensitivity. **D** Heatmap showing the association between expression of core UPR^mt^-related genes and OS in pan-cancer through univariate Cox regression analysis. The result is visualized using the R package ggplot2. The red circle represents a correlation between high expression of gene and poor prognosis in cancer patients, while the blue circle represents a correlation between low expression of gene and poor prognosis.

ATF4 and CHOP are also important bZIP transcription factors but do not contain MTS. ATF4 can induce the expression of CHOP, which dimerizes with another transcription factor C/EBPβ [10]. Notably, the promoters of UPR^mt^-associated genes, such as HSP60, ClpP and mtDNAJ, contain a binding element of CHOP, demonstrating that CHOP participates in the UPR^mt^ [22]. Moreover, ATF4 and CHOP assist ATF5 in activating the UPR^mt^. Under the influence of CHOP and ATF4, ATF5 accumulates in the nucleus during mitochondrial stress and responds to stress through transcriptional programs. The results of the protein-protein interactive network suggest that the interactions among ATF4, ATF5 and CHOP may determine the specificity of UPR^mt^ (Fig. 2A). Since these transcription factors are involved in both the UPR^mt^ and UPR^ER^, revealing the selective role of these mediators will help us to better understand the activation process of the UPR^mt^.

A recent study demonstrates that the v-ATPase/mTORC1 complex located on the surface of lysosomes facilitates cells to process stress signals from mitochondria more efficiently [36]. Specifically, during mitochondrial stress, mTORC1 is activated by v-ATPase on the lysosome surface and then phosphorylates ATF4, ensuring the function of ATF4 in the UPR^mt^. These findings again suggest that UPR^mt^ activation involves interactions between mitochondria and other organelles.

#### Estrogen receptor alpha (ERα) and UPR^mt^

In the mitochondrial intermembrane space (MIS), protein kinase B (PKB)/AKT phosphorylates and activates ERα to regulate the expression of nuclear respiratory factor 1 (NRF1) and mitochondrial protease high-temperature requirement serine protease A2 (HTRA2, also known as OMI) (Fig. 1) [27]. NRF1 binds to SIRT7 and transports SIRT7 to the nucleus to modify chromatin, and HTRA2 degrades misfolded proteins to facilitate cells to cope with crises. In the G93A-SOD1 mouse model of amyotrophic lateral sclerosis (ALS), there is a significant sex difference in the activation of the UPR^mt^ pathway in MIS [37]. Increased expression and proteasome activity of HTRA2 are observed in female mice but not in male mice, which is consistent with ERα being more active in female mice. This coincided with a longer lifespan in female mice and a higher incidence of ALS in men. However, CHOP and HSP60 are not involved in this ERα-dependent process; thus, ERα-involved UPR^mt^ pathways may be independent and complementary to typical UPR^mt^ transcriptional responses.

#### HSF1 and UPR^mt^

HSF1, an important orchestrator of HSR, is indispensable for upregulating the mitochondrial chaperones during UPR^mt^ [38]. Under normal conditions, HSP70 binds to HSF1 and inhibits its activity. Mitochondria stress induces ROS generation and causes the oxidation of DNAJA1 to enhance its binding to HSP70 and other mitochondrial protein precursors, leading to HSF1 release and activation [39]. Therefore, DNAJA1 indirectly activates HSF1 to regulate the expression of ATF5 and mitochondrial chaperones, indicating that HSF1 acts as a signal transfer of oxidative stress to the UPR^mt^ effector. Interestingly, the PP2A-regulated dephosphorylation of HSF1 facilitates the generation of mitochondrial stress-specific variant of HSF1, thereby selectively inducing small HSPs to maintain proteostasis in response to mitochondrial stress in *C. elegans* [40]. The transcription activity of HSF1 is influenced by some interacting proteins to regulate the expression of UPR^mt^ downstream effectors. For example, mitochondrial single-stranded DNA binding protein 1 (SSBP1) binds to HSF1 and modifies chromosomes to increase the transcription of HSP10, HSP60 and HSPA9 to stimulate the UPR^mt^ (Fig. 1) [41]. Interestingly, HSF1 also accumulated in the mitochondria to exacerbate the pathogenesis of Huntington’s disease (HD). Mechanistically, mitochondrial HSF1 induced mitochondrial fragmentation by phosphorylating and activating dynamin-related protein 1 (Drp1), and also impeded mtDNA stabilization and SSBP1 oligomer formation [42]. Moreover, the peptide inhibitor DH1 markedly inhibits the interaction between HSF1 and Drp1 to suppress the mitochondrial localization of HSF1, thus alleviating mitochondrial dysfunction and HD symptoms [43]. In conclusion, HSF1 is involved in UPR^mt^ process through the mediation of expression of mitochondrial function-associated genes. HSF1 activity is regulated by many co-factors, which enables us to better understand the functional network of HSF1 in UPR^mt^.

#### SIRT3 and UPR^mt^

Sirtuins (SIRTs) are NAD^+^-dependent deacetylases and play important roles in the regulation of energy metabolism and stress response, among which SIRT3 is shown to affect mitochondrial metabolism by regulating the UPR^mt^ (for further discussion about SIRT3 and UPR^mt^, see Supplementary file S3).

### UPR^mt^ and cancer

Cancer cells often experience increased mitochondrial stress. Activation of UPR^mt^ guarantees the survival and apoptosis escape of cancer cells. Mitochondrial stress can indeed be both a consequence and a cause of cancer. Various tumorigenesis- and UPR^mt^-related factors (oxidative stress, DNA damage and mutagenesis, increased energy demand and metabolic rewiring) influence and promote each other, forming a positive cycle. A growing body of evidence shows that numerous components of UPR^mt^ widely participate in tumorigenesis.

A recent study reveals that mitochondrial DNA promotes the selective activation of the UPR^mt^ [44]. A significant increase in tumor incidence is observed in mice with UPR^mt^, accompanied by tumor invasion, demonstrating that UPR^mt^ activation exacerbates the occurrence and invasion of tumors. Mechanistically, the pregnancy-associated plasma protein A (PAPP-A)/discoidin receptor 2 (DDR2) pathway is associated with the UPR^mt^ to initiate liver cancer. PAPP-A is a protease involved in the degradation of insulin-like-growth-factor-binding proteins 4/5 (IGFBP4/5) and also acts as an oncogene. PAPP-A could activate DDR2 to promote the invasion and metastasis of cancer cells through the ERK/SNAIL signaling [45].

Additionally, the pan-cancer analysis results demonstrate that the 10 pivotal UPR^mt^-related genes, which have been thoroughly studied in previous literature and selected to be detailed in this review, are associated with overall survival (OS) in certain cancers. Gene Ontology (GO) and Kyoto Encyclopedia of Genes and Genomes (KEGG) analyses suggest that the core UPR^mt^-related genes are significantly enriched in the UPR-associated signaling pathways, such as cellular response to incorrect protein and cellular response to chemical stress (Fig. 2B). Furthermore, these genes are also interlinked with many human diseases, including mitochondrial diseases and cancers (Fig. 2B). More importantly, these key UPR^mt^-related genes are negatively correlated with the sensitivity of chemotherapy drugs according to the GDSC database (Fig. 2C). The above findings all demonstrate that the UPR^mt^ signaling pathway participates in regulating the occurrence and development of tumors through different molecular mechanisms.

### Relationships between UPR^mt^-related proteins and oncogenesis

#### The roles of ATF5 in oncogenesis

**Fig. 3:**
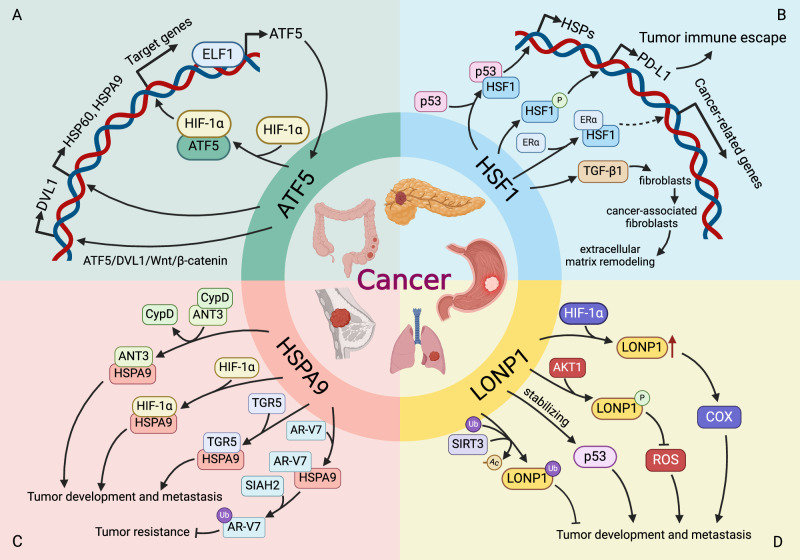
The components of UPR^mt^ affect oncogenesis through various signaling pathways. **A** ATF5 regulates the expression of tumorigenic genes, such as HSP60. ATF5 binds to HIF-1α to regulate the transcription of target genes. These downstream genes enhance the proliferation, migration and invasion of cancer cells. **B** Phosphorylated HSF1 plays a role in tumor immune escape by modulating the expression of HSPs and PD-L1. HSF1 also interacts with p53 and ERα to regulate gene expression to facilitate oncogenesis. **C** HSPA9 interacts with ANT3, HIF-1α or TGR5 to promote tumor development and metastasis. HSPA9 also interacts with AR-V7 to enhance ubiquitination and degradation of AR-V7 to overcome drug resistance. **D** LONP1 is deacetylated by SIRT3 and degraded by ubiquitination, thus preventing tumor development. LONP1 inhibits apoptosis by stabilizing p53. LONP1 attenuates the generation of ROS or facilitates cancer cells to adapt to anoxic environment under the influence of AKT1 or HIF-1α. Figure was generated utilizing Biorender.com.

ATF5 is a key regulator of the UPR^mt^ in mammals and is involved in the tumorigenesis of a variety of cancers [46–48]. ATF5 is dramatically overexpressed and correlated with poorer prognosis in many cancer patients (Fig. 2D) [49]. As an upstream regulator, E74-like ETS transcription factor 1 (ELF1), playing diverse roles in lymphocyte development, angiogenesis and cancer, stimulates ATF5 gene transcription by directly binding to its promoter (Fig. 3) [50]. Moreover, ATF5 overexpression is strongly linked with both clinicopathological characteristics and relapse-free survival rates in bladder urothelial carcinoma patients. Mechanistically, ATF5 promotes the transcription of disheveled-1 (DVL1), a potent activator of Wnt/β-catenin, and increases tumor sphere formation ability through the ATF5/DVL1/Wnt/β-catenin axis (Fig. 3) [51]. ATF5 knockdown suppresses the proliferation, migration and invasion abilities of esophageal cancer cells. ATF5 can bind to hypoxia-inducible factor 1α (HIF-1α), an oxygen-sensing transcriptional regulator orchestrating a complex of adaptive cellular responses to hypoxia and oncogenesis, to act as a coactivator and form a transcription complex, thereby regulating the expression of HIF-1α target genes (Fig. 3) [52]. Decreased ATF5 expression inhibits angiogenesis and tumor growth in vivo [53]. Additionally, activation of Maf1, a master repressor of Pol III-dependent transcription and mechanistic target of rapamycin (mTOR) downstream effector, alleviates ionizing radiation-induced UPR^mt^ in lung cancer cells. ATF5 is needed for this process, as evidenced by the fact that silencing ATF5 greatly reduced the Maf1 inhibition-induced upregulation of HSP60 and HSPA9, suggesting that Maf1 modulated the UPR^mt^ in an ATF5-dependent manner [54]. In summary, ATF5 not only elevates the expression of antiapoptotic genes but also increases the levels of factors that regulate growth and metabolism, thereby contributing to radiotherapy resistance and promoting tumor cell invasion [8, 55, 56].

#### The roles of ATF4 in oncogenesis

Dysregulation of ATF4 expression has been implicated in various types of cancer [57–59]. Higher ATF4 expression is significantly correlated with worse overall survival in gastric cancer patients, indicating that ATF4 is an independent prognostic factor [60]. Silencing ATF4 strongly blocks cell proliferation, invasion, migration and cell cycle progression and increases drug sensitivity, possibly by modulating autophagy and asparagine metabolism [61]. Inhibition of glutaminolysis upregulates and activates ATF4 by reducing FTO-mediated m6A modification and YTHDF2-mediated mRNA degradation, ultimately positively regulating CHOP transcription to trigger autophagy [62, 63]. Targeting ATF4-regulated protective autophagy enhances the anticancer effect of glutaminolysis inhibition [64]. In pancreatic ductal adenocarcinoma (PDAC), TGF-β1 secreted from cancer-associated fibroblasts (CAFs) drives the elevation of ATF4 expression through the SMAD2/3 pathway to modulate cancer cell proliferation, migration and stemness. ATF4 then directly promotes the expression of multidrug resistance protein 1 (MRP1, also known as ABCC1) by binding its promoter, leading to gemcitabine resistance in PDAC [65]. Depletion of ATF4 also decreases metastasis and tumor growth in breast cancer by mediating the TGF-β/SMAD and mTOR/RAC1-RHOA pathways [66]. The EWS-FLI1 fusion protein directly activates ATF4 transcription by binding to its promoter. ATF4 then controls the transcription of target genes involved in the core serine synthesis pathway in Ewing sarcoma [67]. Kristen rat sarcoma (KRAS), the most common mutated oncogene in human cancers, cooperates with NRF2 to upregulate ATF4 expression during nutrient stress, thereby modulating the amino acid uptake and asparagine biosynthesis in lung cancer [68]. Asparagine synthetase (ASNS) is an important target gene of ATF4 that modulates protein synthesis, promotes tumor growth and suppresses cell death [69]. Inhibition of ASNS sensitizes lung cancer cells to L-asparaginase, a cornerstone drug in the treatment of acute lymphoblastic leukemia (ALL). Ribosomal protein L41 (RPL41) is a tumor suppressor and its downregulation and deletions are frequently detected in human cancers [70]. RPL41 promotes ATF4 translocation to the cytoplasm, increases the ATF4 phosphorylation, thus accelerating ATF4 degradation and sensitizing lung cancer and retinoblastoma cells to chemotherapeutic drugs [71]. Moreover, the eIF2α/ATF4 axis is associated with radioresistance through the modulation of glutathione biosynthesis and ROS generation in breast cancer [57]. Elevated ATF4 expression also correlates with resistance to multiple DNA-interacting drugs. For instance, cisplatin upregulates ATF4 expression, and ATF4-deficient cancer cells exhibit elevated sensitivity to cisplatin-induced cell death [72]. Mechanistically, ATF4 promotes multidrug resistance by directly regulating the transcription of SIRT1 and STAT3 [73]. The circadian transcription factor Clock promotes the transcription of ATF4 following cisplatin treatment, thereby mediating drug resistance by regulating the genes for glutathione metabolism [74]. YAP and TAZ are transcriptional coactivators in Hippo signaling cascade and promote cell proliferation, tissue regeneration and tumorigenesis. Notably, YAP/TAZ inhibits ferroptosis in liver cancer and is associated with sorafenib resistance. Activated YAP/TAZ promotes ATF4 nuclear localization, increases its transcriptional activity and cooperates with ATF4 to induce SLC7A11 transcription to overcome sorafenib-triggered ferroptosis [75]. Together, ATF4 coordinates and integrates various cellular processes by regulating different genes to facilitate tumorigenesis and chemoresistance.

#### The roles of HSF1 in oncogenesis

HSF1 not only maintains proteostasis by inducing HSP expression in response to stresses but also regulates tumorigenesis through multiple network pathways and target genes [76–78]. An increasing number of studies has summarized that HSF1 achieves its cancer-promoting effects by inhibiting cell apoptosis, accelerating cell proliferation and migration, reprogramming metabolic programs, and supporting the tumor microenvironment [79, 80]. Consistent with its oncogenic role, HSF1 is overexpressed or overactivated in a broad spectrum of cancers and negatively correlated with the prognosis of cancer patients (Fig. 2D) [81]. The pan-cancer analysis of HSF1 reveals that HSF1 is involved in diverse cancer-associated signaling pathways, and is closely associated with immune cell infiltration and efficacy of immunotherapy [78, 82]. PIM2-regulated phosphorylation of HSF1 at Thr120 regulates PD-L1 expression by binding to its promoter and facilitates oncogenesis (Fig. 3) [82]. HSF1 is also activated in stromal fibroblasts, a component of the tumor microenvironment (TME), leading to remodeling of the extracellular matrix that promotes colon cancer development [83]. Moreover, thrombospondin 4 (TSP-4) secreted from CAFs binds to integrin α2 to promote HSF1 phosphorylation at Ser326 to support the malignant phenotypes of gallbladder cancer cells, including cell proliferation, epithelial-mesenchymal transition (EMT) and cancer stemness [84]. Activated HSF1 further controls the expression of TGF-β1 to help the transdifferentiation of fibroblasts into CAFs, thereby forming positive feedback [84]. HSF1 interacts with ERα and cooperates to regulate some common genes in breast cancer (Fig. 3) [85]. Moreover, HSF1 is a target of FTO-regulated and YTHDF2-dependent m6A modification to accelerate the development and progression of multiple myeloma [86]. Additionally, the ABL2/HSF1/E2F axis is needed for the brain metastasis of lung cancer [87]. The ERK/FBXW7/HSF1/MDR1 pathway is closely linked with chemoresistance [88]. Inhibition of HSF1 significantly overcomes resistance to epidermal growth factor receptor tyrosine kinase inhibitors (EGFR-TKIs), which is the standard treatment for NSCLC patients with EGFR mutations, by downregulating the expression of HSPs [89].

Numerous high-throughput screening studies have identified many molecules that act as HSF1 inhibitors [81, 90, 91]. However, the search for drugs targeting HSF1 is challenging due to the broad roles of transcription factors and the lack of specificity of small molecules. However, targeting the upstream regulatory factors or signaling pathways of HSF1 for drug screening provides an indirect pharmacological strategy to more easily inhibit the tumor-promoting effect of HSF1. Notably, combining HSF1-targeted cancer therapy with other chemotherapeutic agents and treatment strategies has become an effective oncology strategy and has been initially validated in several studies [92].

#### The roles of HSPA9 in oncogenesis

HSPA9, also called mitochondrial HSP70/GRP75/mortalin, is a survival-promoting protein and plays an important role in mitochondrial function. Recent literature has demonstrated that HSPA9 expression is upregulated and associated with poor prognosis in many types of cancer patients (Fig. 2D) [93–95]. Additionally, circulating HSPA9 levels are elevated in colorectal cancer patients and correlated with worse prognosis, indicating that HSPA9 is a useful prognostic biomarker [96–99]. HSPA9 not only modulates the Raf/MEK/ERK signaling pathway but also promotes the PP1α-MER1/2 interaction to increase MEK1/2 dephosphorylation in KRAS and BRAF mutant cancer cells [100, 101]. Moreover, HSPA9 blocks the degradation of high mobility group A1 (HMGA1) and promotes the activation of the HMGA1/JNK/c-Jun axis in lung cancer to stimulate cancer cell growth and metastasis [102]. In breast cancer, HSPA9 facilitates oncogenesis through the Wnt/GSK3β/β-catenin pathway [103]. Thus, knockdown of HSPA9 markedly suppresses cell proliferation, inhibits EMT, induces cell cycle arrest and initiates cell death in various cancer cells [104]. In vemurafenib-resistant BRAF-mutant melanoma cells, HSPA9 depletion triggers cell death in a MEK/ERK- and ANT/CypD-dependent manner, indicating that HSPA9 is an effective target in drug-resistant tumors [105]. With the gradual identification of HSPA9-interacting proteins, the molecular mechanism of HSPA9-regulated oncogenesis has gradually emerged. Proteomic analysis identifies adenine nucleotide translocase 3 (ANT3) as an interacting protein of HSPA9 and indicates that HSPA9 can disrupt the interaction between ANT3 and cyclophilin D (CypD) in BRAF mutant cancer cells (Fig. 3) [106]. In prostate cancer, HSPA9 maintains the protein stability of sine oculis homeobox 1 (SIX1), a developmental transcriptional regulator frequently overexpressed in human cancers, by inhibiting its polyubiquitination and degradation through recruiting the deubiquitinating enzyme ubiquitin-specific protease 1 (USP1). Either genetic or pharmacological inhibition of the HSPA9-SIX6-USP1 complex effectively impedes progression of prostate cancer (PC) and castration resistance [107]. Androgen receptor splice variant 7 (AR-V7) is a form of ligand-independent and constitutively activating variant of androgen receptor (AR), and plays an important role in castration-resistant PC. The HSPA9-AR-V7 complex can recruit the E3 ligase SIAH2 to enhance the ubiquitination and degradation of AR-V7 to overcome drug resistance in PC (Fig. 3) [108]. Moreover, HSPA9 also interacts with HIF-1α to promote the association of HIF-1α with voltage-dependent anion channel 1 (VDAC1) and hexokinase II (HK-II) in the mitochondrial outer membrane, thereby enhancing resistance to apoptosis under hypoxic conditions [109]. HSPA9 is also associated with cytoplasmic sequestration and retention of p53 [110, 111]. Furthermore, Takeda-G-protein receptor-5 (TGR5) accelerates cancer cell proliferation and migration and promotes cell death resistance partially by interacting with HSPA9. Knockdown of HSPA9 reversed various oncogenic phenotypes mediated by TGR5 [112].

**Fig. 4:**
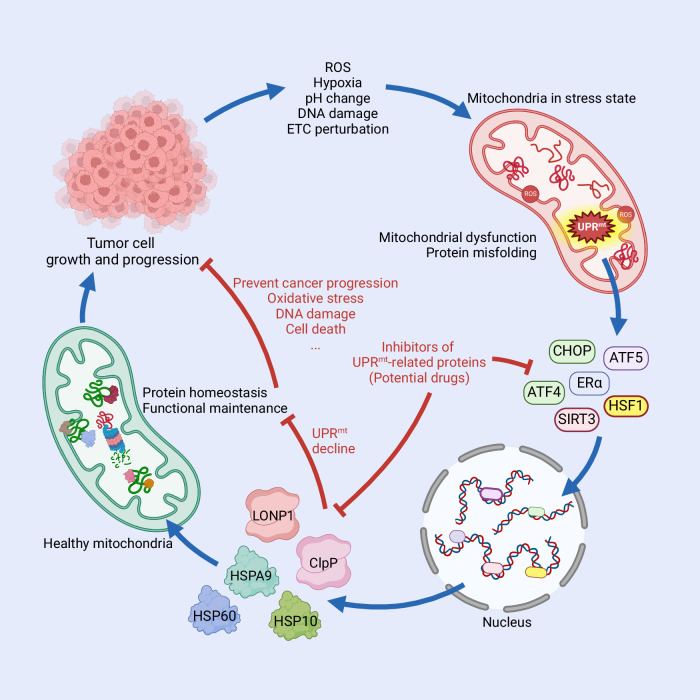
Relationship between UPR^mt^ and oncogenesis. With the progression of tumor, cancer cells face problems such as increased ROS levels and hypoxia, which puts mitochondria in a state of stress, manifested as mitochondrial dysfunction and protein misfolding. Meanwhile, UPR^mt^ is stimulated. ATF5 and other UPR^mt^ regulators transmit signals between mitochondria and nucleus, and promote the transcription of UPR^mt^-related genes. Transcribed mitochondrial chaperonins and proteases enter mitochondria to facilitate proteins to fold correctly or eliminate misfolded proteins, allowing the mitochondria to restore proteostasis and normal function. Inhibitors of UPR^mt^-related proteins suppress the activity of upstream UPR^mt^ regulators or downstream functional proteins, thus making it difficult for mitochondria to recover healthy state and ultimately accelerating cancer cell death. Therefore, these inhibitors of UPR^mt^ are considered as potential drugs for cancer treatment. Figure was generated utilizing Biorender.com.

The above literature suggests that the development of small-molecule compounds or drugs targeting HSPA9 is a promising strategy for cancer therapy. MKT-077, a specific inhibitor of HSPA9, exhibits anticancer effects by inducing apoptosis and necrosis and reducing resistance to oxidative stress in bladder cancer (Table 1 and Fig. 4) [113]. Additionally, several analogs of MKT-077, such as JG-98, JG-194 and JG-231, have been synthesized to bind to HSPA9 and suppress cancer cell growth, thereby enhancing the clinical translational value of MKT-077 (Table 1) [114, 115].

**Table 1:** Compounds targeting UPR^mt^ for cancer therapy.

Target	Compounds	Mechanism	Effects	Reference
HSPA9	MKT-077	Bind to HSPA9, thus inhibiting the HSPA9-p53 interaction and releasing p53	Reduce cell viability Induce apoptosis and necrosis Reduce resistance to oxidative stress	[113]
HSPA9	MKT-077 analogs JG-98, JG-194 and JG-231	Bind to HSPA9	Inhibit the growth of tumor cells Induce apoptosis	[114, 115]
HSP60	KHS101	Bind to and inhibit the folding activity of HSP60	Disrupt energy metabolism Suppress cell proliferation	[122]
HSP60	Doxorubicin	Promote HSP60 acetylation and disrupt the HSP60-p53 interaction Promote the ubiquitination and degradation of HSP60	Inhibit the growth of tumor cells Induce cell cycle arrest Trigger the senescent phenotype Induce apoptosis	[123]
HSP60	Bis-aryl-α,β-unsaturated ketone	Bind to HSP60	Inhibit cell clonogenicity Reduce cell migration	[124]
HSP60	Benzimidazole Benzimidazole-2-ones	Bind to HSP60	Inhibit HSP60 Exhibit anti-inflammatory and anti-cancer properties	[126]
HSP60	curcumin	Reduce the HSP60 S-nitrosylation Decrease HSP60 expression	Suppress cell proliferation Induce apoptosis	[125, 127]
LONP1	CDDO, CDDO-Me, CDDO-Im and CDDO-anhydrid	CDDO, CDDO-Me and CDDO-Im directly bind with LONP1 and inhibit the atpase and protease activities of LONP1 CDDO-anhydride competitively inhibits the atpase activity of LONP1	Induce cytotoxicity Induce apoptosis	[140]
LONP1	Obtusilactone A (OA) and (-)-Sesamin	Bind to LONP1 Cause DNA double-strand breaks Affect the JNK pathway	Induce cytotoxicity Induce apoptosis	[141]
ClpP	inhibitors TG42 and TG53	Inhibit ClpP proteolytic activity	Reduce cell migration Induce apoptosis	[153]
ClpP	agonist ONC201	Bind to and overactivate ClpP to induce the degradation of respiratory chain complex subunits	Suppress cell proliferation Induce apoptosis Induce cell cycle arrest Reduce cell Invasion	[150, 155]
ClpP	ONC201 analogs (TR compounds, IMP075 and ZG111)	Bind to and overactivate ClpP to induce the degradation of respiratory chain complex subunits	Cause mitochondrial dysfunction Induce apoptosis Prevent cancer cell adhesion and invasion Suppress cell proliferation Induce cytotoxicity	[150, 154, 156, 157]

#### The roles of HSP60 in oncogenesis

HSP60 (HSPD1), another important mitochondrial chaperone, performs essential tasks for maintaining mitochondrial proteostasis. In breast cancer, accumulated misfolded mitochondrial proteins activate the UPR^mt^ and upregulate HSP60 and HSP10 expression. Altered HSP60 expression is associated with a variety of cancers, serving as a tool for diagnosis and prognosis of cancer patients (Fig. 2D) [116, 117]. Elevated HSP60 expression activates the ERK1/2 pathway and promotes the growth of adenocarcinoma cells [118]. Moreover, HSP60 upregulates the proto-oncogene MYC and impedes the function of the tumor suppressor p53, thereby allowing cancer cells to grow and invade surrounding tissues [119]. HSP60 downregulation increases the intracellular ROS levels and activates the AMPK-mTOR pathway, thereby inhibiting the proliferation of glioblastoma cells [120].

A number of HSP60 inhibitors have been identified which can interact with HSP60 or alter its post-translational modifications (Table 1) [121]. For example, the synthetic small-molecule KHS101 disrupts energy metabolism in glioblastoma and NSCLC by inhibiting the folding activity of HSP60 [122]. Doxorubicin, a medication used to treat various cancers, binds to HSP60 to promote its acetylation, ubiquitination and degradation (Table 1) [123]. HSP60 inhibitors represent a promising avenue in cancer research by targeting the protein folding machinery of cancer cells, potentially leading to cell death and reduced tumor growth (Table 1) [124–127].

#### The roles of LONP1 in oncogenesis

LONP1 and ClpP are the two major ATP-dependent proteases in mammals, involved in regulating protein assembly, folding and degradation, as well as controlling mitochondrial function under stress conditions [128]. Mitochondrial proteotoxic stress caused by loss of LONP1 activates the UPR^mt^ via ATFS-1 in *C. elegans* [129]. LONP1 is a multifaceted enzyme with different functions, including proteolysis, chaperone activity and binding of mtDNA [130, 131]. LONP1 is upregulated by several stress stimuli. LONP1 degrades not only misfolded or damaged proteins but also apoptosis- and metabolism-related proteins, thus helping to protect the mitochondria from oxidative damage [132, 133]. LONP1 is activated in various human cancer tissues, and its overexpression is associated with prognosis in multiple types of cancer (Fig. 2D) [134]. As one of the molecular mechanisms, LONP1 is transcriptionally upregulated by CHOP and CEB/P, two UPR^mt^ signature proteins [132]. Moreover, LONP1 is preferentially mediated by the p53 and β-catenin. Knockdown of LONP1 inhibits cervical cancer cell proliferation, migration and invasion by influencing mitophagy and autophagy [121]. LONP1 is involved in metabolic reprogramming in cancer cells by remodeling the OXPHOS complex. When cells lack LONP1, severe mitochondrial dysfunction occurs. However, high level of LONP1 can induce a shift from oxidative respiration to glycolytic metabolism [128, 135]. LONP1 promotes the transformation of metabolic mode to glycolysis during carcinogenesis to provide a large amount of energy for rapid cell proliferation [130]. LONP1 is deacetylated by SIRT3 at the K145, making it more susceptible to ubiquitination and degradation (Fig. 3) [136]. With increasing age, the loss of SIRT3 increases the degree of acetylation of LONP1, which promotes the occurrence and progression of tumors. Oxidative stress also induces LONP1 expression, which in turn promotes the proliferation and migration of cancer cells [130]. Under hypoxic conditions, AKT1 phosphorylates LONP1 at Ser173 and Ser181 to enhance its protease activity, which attenuates ROS effects and promotes cancer cell migration (Fig. 3) [137]. Thus, interfering with this phosphorylation process inhibits tumor growth and metastasis. During anoxia, LONP1 is upregulated by HIF-1α, and its enzyme activity is enhanced by the cytochrome c oxidase (COX), thereby facilitating cancer cell adaptation to anoxic environments (Fig. 3) [132]. Additionally, LONP1 inhibits apoptosis by stabilizing p53 in extreme environments, and cells with p53 deletion have stronger proliferation ability under the influence of LONP1 [130]. LONP1 ablation also blocks tumor metastasis by inhibiting EMT and extracellular matrix remodeling through the c-Jun N-terminal kinase (JNK) pathway in human pancreatic cancer [138]. More importantly, LONP1 participates in ferroptosis by regulating the peroxidase GPX4 and NRF2/Keap1 signaling pathways, providing a cancer treatment strategy via ferroptosis [139]. LONP1 also assists cancer cell migration and invasion by promoting CAFs formation in the TME and leads to PD-L1-mediated immune escape [134].

Under the conditions of artificial induction of colon carcinoma, *Lonp1*^*+/–*^ mice show lower tumor incidence and milder symptoms than wild-type mice. LONP1 is also essential for the metastasis of cancer cells in vivo. Silencing LONP1 results in a significant reduction in the melanoma cell metastases [128].

Several chemical inhibitors have the potential to inhibit LONP1 activity to prevent cancer progression and sensitize cancer cells to chemotherapy (Table 1 and Fig. 4). The synthetic oleanane 2-cyano-3,12-dioxooleana-1,9-dien-28-oic acid (CDDO), obtusilactone A (OA) and (-)-Sesamin directly bound to LONP1 to inhibit its activity (Table 1) [140, 141]. Furthermore, overexpression of LONP1 can salvage the inhibition of proteasome activity by proteasome inhibitors; thus, therapeutic strategies targeting LONP1 may affect the effectiveness of existing treatment modalities (Table 1 and Fig. 4) [142].

#### The roles of ClpP in oncogenesis

ClpP is an ATP-dependent protease in the mitochondrial matrix that degrades misfolded or denatured proteins [143]. ClpP is overexpressed in many human cancers and associated with poor prognosis in breast and lung cancer [144]. ClpP expression in metastatic lesions is higher and affects the proliferation and metastasis of tumor cells [145]. Inhibition of ClpP suppresses the proliferation, migration and invasion of cancer cells, which is manifested by a blocked cell cycle and a lower degree of metastasis [146]. Moreover, ClpP inhibition also triggers oxidative stress, which subsequently decreases the expression of the cell motility effector caveolin-1, resulting in impaired energy metabolism and reduced metastatic potential [147]. In breast cancer, silencing ClpP decreases the activity of SRC and AKT and phosphorylation of PI3K, thereby interfering with the proliferation, migration and apoptosis of breast cancer cells [148]. However, overexpression of ClpP in ovarian cancer cells reduces cell motility and represses cell migration and invasion by inducing mitochondrial respiratory chain disorder [149]. Activation of ClpP also prevents cancer cell growth by disrupting mitochondrial structure and function, and causes cell death in a p53-independent manner [146, 150]. Therefore, too low or too high levels of ClpP lead to cancer cell death through different mechanisms in different cancers.

ClpP expression is markedly associated with drug resistance, and high expression of ClpP reduces apoptosis during chemotherapy, while silencing ClpP improves chemotherapeutic response [146]. ClpP enhances resistance to cisplatin in ovarian cancer cells by suppressing mitophagy and exacerbating cellular stress [151]. Thus, targeting ClpP to increase the drug sensitivity is beneficial for cancer treatment [152].

Drugs developed based on ClpP can achieve the ultimate goal of treating cancer by modulating ClpP activity (Table 1 and Fig. 4). For example, TG42 and TG53, modified analogs of the first ClpP inhibitor trans-β-lactones, inhibit ClpP proteolytic activity, reduce cell migration and induce apoptosis in liver cancer cells (Table 1) [145, 153]. Interestingly, some ClpP agonists were also identified. The small molecule ONC201 and its analogs activate ClpP to disrupt respiratory chain integrity, thus affecting mitochondrial function and exhibiting anticancer properties (Table 1) [150, 154–157]. Since the effect of ClpP on cancer cells is closely associated with its expression level, whether increasing or inhibiting ClpP activity is more effective in treating cancer needs to be further explored (Fig. 4).

## Conclusions and perspective

The UPR^mt^, triggered by multiple mitochondrial stresses, is a vital process by which mitochondria ensure the correct folding and homeostasis of proteins. With the continuous understanding of the UPR^mt^, the molecular mechanism model of the UPR^mt^ is gradually being constructed and improved, providing a solid foundation for its further application in disease treatment. However, the undisclosed parts of its triggering mechanism have also drawn the attention of researchers. Additionally, it is not clear whether there is an interaction between several seemingly independent pathways that stimulate the UPR^mt^ and whether one pathway is dominant in certain cancer cells. Furthermore, many examples of cellular non-autonomous UPR^mt^ have been found, but the differences in mechanism, excitation and consequences between cell-induced and non-autonomous UPR^mt^ are still unclear. We wonder whether a centain type of cancer cell is more inclined to rely on the UPR^mt^ for survival. However, the possible side effects of UPR^mt^ on healthy cells should be noted.

**Fig. 5:**
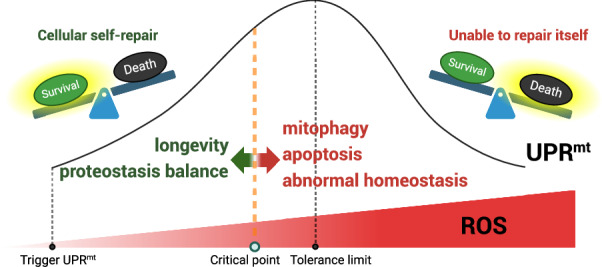
UPR^mt^ is a double-edged sword. When the stress faced by cells reaches a certain level, UPR^mt^ is triggered. Within a certain range, cells repair themselves, which helps maintain proteostasis and survive. A certain degree of UPR^mt^ can prolong lifespan, showing beneficial effects on organisms. The activation of UPR^mt^ intensifies as the cells are subjected to increased stress. Up to a certain point, the effects of UPR^mt^ on cells are reversed, and high level of UPR^mt^ have some negative effects on cells, such as protein dyshomeostasis, mitophagy and apoptosis. However, the mechanisms by which cells perceive and judge stress intensity are still unclear, and the adverse effects of excessively high level of UPR^mt^ on cells need to be explored. More importantly, under what circumstances do cells choose to abandon self-rescue and initiate apoptosis, as well as the mechanisms that play a role in this critical turning point require further research. Figure was generated utilizing Biorender.com.

The UPR^mt^, as an emergency measure taken by mitochondria, is of great significance in maintaining the growth and proliferation of cancer cells (for UPR^mt^ in cancer treatment, see Box 2) (Fig. 4). However, the UPR^mt^ is a complex process with dual roles. Faced with a certain amount of protein misfolding or ROS, cells activate the UPR^mt^ to maintain intracellular proteostasis and help cells survive. However, when exposed to excessive stress, mitochondria initiate mitophagy and even apoptosis [158]. Moderate UPR^mt^ activation exhibits the potential for treating neurodegenerative diseases and heart disease, but excessive activation of the UPR^mt^ promotes cardiomyocyte apoptosis, the death of dopaminergic neurons, and mitochondrial dysfunction in the ALS mouse model [159]. These findings suggest that there may be crossover signaling pathways between the UPR^mt^ and cell death pathways. These phenomena have led us to realize that the benefits of the UPR^mt^ appear to be variable and fragile (Fig. 5). For a period of time after mitochondrial stress, cells continue to enhance UPR^mt^ to save themselves. However, when the stress intensity reaches the maximum tolerance limit, cells may clear the damaged mitochondria through mitophagy to ensure the survival of the entire cell (Fig. 5). If the cells cannot maintain the high activation of UPR^mt^ to save themselves, a large number of cells will suffer cell death (Fig. 5). This model we predict has been described in the UPR^ER^ [160, 161]. Although more detailed studies are lacking, overactivation of UPR^mt^ has more complex and multi-dimensional effects, which may be an effective therapeutic strategy for cancer (Box 2). To this end, more sensitive detection methods and biomarkers of the UPR^mt^ may be helpful.

In conclusion, UPR^mt^, as a mitochondrial stress mechanism, is highly associated with tumor progression and has great potential for clinical application (Box 2). The UPR^mt^ also affects aging and many other human diseases; thus, targeting the UPR^mt^ is a relatively new and promising therapeutic strategy [162].

Box 2 Strategies for targeting UPR^mt^ in cancer therapy Targeting the UPR^mt^ has emerged as a promising strategy in cancer treatment. Firstly, the UPR^mt^ could serve as diagnostic or prognostic biomarker for cancer patients. Recently, a new UPR^mt^-based signature has been successfully developed to classify HCC patients into distinct subtypes with different clinical features, prognosis, drug sensitivities and response to immunotherapy, aiding in the treatment selection and patient management. Secondly, inhibiting specific components (such as HSP60 and HSPA9) of the UPR^mt^ disrupts the mitochondrial function in cancer cells, leading to reduced tumor growth and survival. Thirdly, suppression of UPR^mt^ signaling prevents cancer cells from adapting to mitochondrial stress caused by chemotherapeutic agents, making them more susceptible to cell death. Furthermore, combining UPR^mt^-targeted therapies with existing treatments, such as chemotherapy or immunotherapy, provides a synergistic effect, improving treatment outcomes by attacking cancer cells from multiple angles.

## Supplementary information

Supplementary File 1

Supplementary File 2

Supplementary File 3
