# Supplementary material for: A bird’s eye view of mitochondrial unfolded protein response in cancer: mechanisms, progression and further applications
Source: Cell Death Dis. 2024 Sep 11;15(9):667. doi: 10.1038/s41419-024-07049-y (PMC11390889; doi:10.1038/s41419-024-07049-y)
Supplement: Supplementary file 1 [file 41419_2024_7049_MOESM1_ESM.doc]

**Supplementary File 1. The relationship between UPR^ER^ and UPR^mt^.**

Because cell organelles are physically and functionally associated, and consequently, their stress responses are often intertwined. UPR^ER^ is triggered by the accumulation of unfolded and/or misfolded proteins in the ER, and activates an adaptive UPR through inositol-requiring enzyme-1 (IRE1), double-stranded RNA-activated protein kinase (PKR)-like ER kinase (PERK), and activating transcription factor-6 (ATF6) [1-3]. In contrast, UPR^mt^ is induced when proteostasis is broken with an accumulation of misfolded and unfolded proteins in the mitochondria and regulated by ATF5, ATF4, CHOP, HSF1 and other factors in mammals [4-6].

Many pieces of evidence suggest that UPR^ER^ and mitochondrial stress pathways are intertwined. For example, ATF4 and CHOP are classical downstream inducers during ER stress [7]. Meanwhile, robust translation of ATF4 promotes CHOP transcription and consequently, ATF4 and CHOP upregulate ATF5 expression, a central player in UPR^mt^. In mammalian cells, ATF5, ATF4, and CHOP are all required for UPR^mt^ induction [8, 9]. It is known that ER stress can result in mitochondrial dysfunction, mitochondrial oxidative stress, and trigger inflammation through some mechanisms, such as ER-to-mitochondria calcium transfer and inhibition of PERK-caused mitophagy [10, 11]. A recent study has revealed that ER stress induces IRE1 kinase activity-dependent upregulation of sphingosine 1-phosphate (S1P), leading to enhanced UPR^mt^ signaling [11].

In contrast, mitochondria stress also spreads to the ER. For example, mitochondrial translation inhibitor (doxycycline) and mitochondrial toxin (paraquat) not only induce UPR^mt^ but also activate UPR^ER^ signaling pathway [11]. Additionally, the expression of the mitochondrial protease LONP1 is markedly induced in a PERK-dependent manner by ER stress, suggesting that UPR^ER^ could directly affect mitochondrial proteostasis [12, 13]. Moreover, ER-mitochondria membrane contacts, also known as MAMs (mitochondria-associated membranes), are specialized regions where the ER and mitochondria are closely apposed, allowing for direct communication and exchange of metabolites and signaling molecules between these two organelles. MAMs play a crucial role in various cellular processes, including calcium signaling, lipid metabolism and apoptosis, to sustain healthy collaboration [14, 15]. In summary, UPR^ER^ and UPR^mt^ are precisely coordinated, not only requiring close communication with the nucleus, but also influencing each other.

**References**

1. Chen X, Shi C, He M, Xiong S, Xia X. Endoplasmic reticulum stress: molecular mechanism and therapeutic targets. Signal Transduct Target Ther. 2023;8(1):352.

2. Hetz C. The unfolded protein response: controlling cell fate decisions under ER stress and beyond. Nat Rev Mol Cell Biol. 2012;13(2):89-102.

3. Hetz C, Zhang K, Kaufman RJ. Mechanisms, regulation and functions of the unfolded protein response. Nat Rev Mol Cell Biol. 2020;21(8):421-38.

4. Inigo JR, Chandra D. The mitochondrial unfolded protein response (UPR(mt)): shielding against toxicity to mitochondria in cancer. J Hematol Oncol. 2022;15(1):98.

5. Uoselis L, Lindblom R, Lam WK, Küng CJ, Skulsuppaisarn M, Khuu G, et al. Temporal landscape of mitochondrial proteostasis governed by the UPR(mt). Sci Adv. 2023;9(38):eadh8228.

6. Zhou Z, Fan Y, Zong R, Tan K. The mitochondrial unfolded protein response: A multitasking giant in the fight against human diseases. Ageing Res Rev. 2022;81:101702.

7. Walter P, Ron D. The unfolded protein response: from stress pathway to homeostatic regulation. Science. 2011;334(6059):1081-6.

8. Costa-Mattioli M, Walter P. The integrated stress response: From mechanism to disease. Science. 2020;368(6489).

9. Keerthiga R, Pei DS, Fu A. Mitochondrial dysfunction, UPR(mt) signaling, and targeted therapy in metastasis tumor. Cell Biosci. 2021;11(1):186.

10. Onat UI, Yildirim AD, Tufanli Ö, Çimen I, Kocatürk B, Veli Z, et al. Intercepting the lipid-induced integrated stress response reduces atherosclerosis. J Am Coll Cardiol. 2019;73(10):1149-69.

11. Yildirim AD, Citir M, Dogan AE, Veli Z, Yildirim Z, Tufanli O, et al. ER Stress-induced sphingosine-1-phosphate lyase phosphorylation potentiates the mitochondrial unfolded protein response. J Lipid Res. 2022;63(10):100279.

12. Han J, Back SH, Hur J, Lin YH, Gildersleeve R, Shan J, et al. ER-stress-induced transcriptional regulation increases protein synthesis leading to cell death. Nat Cell Biol. 2013;15(5):481-90.

13. Hori O, Ichinoda F, Tamatani T, Yamaguchi A, Sato N, Ozawa K, et al. Transmission of cell stress from endoplasmic reticulum to mitochondria: enhanced expression of Lon protease. J Cell Biol. 2002;157(7):1151-60.

14. Barazzuol L, Giamogante F, Calì T. Mitochondria associated membranes (MAMs): Architecture and physiopathological role. Cell Calcium. 2021;94:102343.

15. Rodríguez-Arribas M, Yakhine-Diop SMS, Pedro JMB, Gómez-Suaga P, Gómez-Sánchez R, Martínez-Chacón G, et al. Mitochondria-associated membranes (MAMs): Overview and its role in Parkinson's disease. Mol Neurobiol. 2017;54(8):6287-303.
