# Supplementary material for: A bird’s eye view of mitochondrial unfolded protein response in cancer: mechanisms, progression and further applications
Source: Cell Death Dis. 2024 Sep 11;15(9):667. doi: 10.1038/s41419-024-07049-y (PMC11390889; doi:10.1038/s41419-024-07049-y)
Supplement: Supplementary file 2 [file 41419_2024_7049_MOESM2_ESM.doc]

**Supplementary File 2. Cell non-autonomous UPR^mt^.**

Because of the existence of cell non-autonomous UPR^mt^, when one tissue is stressed, different tissues will face survival pressure together as a regulated whole. This can help organisms cope with adverse environments to some extent, but when the UPR^mt^ is excessively stimulated, it may have more serious consequences.

Mitokines, such as fibroblast growth factor 21 (FGF21) and growth differentiation factor 15 (GDF15), are signaling molecules that facilitate the cell non-autonomous UPR^mt^ [1-3]. These molecules are crucial for systemic regulation and can have various effects on the organism’s adaptation to stress and potentially extend lifespan [4]. FGF21 and GDF15 are induced by mitochondrial stress and regulated by ATF4 and CHOP, key components of the UPR^mt^ [5]. FGF21 is a hormone produced in the liver and adipose tissues and can enhance insulin sensitivity, promote fatty acid oxidation, and reduce body weight [6]. FGF21 functions as a mediator of the UPR^mt^ to help alleviate mitochondrial stress by inhibiting the synthesis of new proteins and promoting the degradation of damaged proteins [7]. FGF21 also induces the expression of genes involved in mitochondrial biogenesis and repair, which may help restore mitochondrial function [1, 7]. GDF15, also known as macrophage inhibitory cytokine 1 (MIC-1), is produced in response to a wide range of physiological and pathological stresses [4]. GDF15 is associated with the regulation of energy balance, inflammation and cancer. GDF15 plays a role in reducing food intake and body weight, which may be a protective mechanism to decrease energy demand and mitigate mitochondrial stress [6]. Strikingly, elevated levels of FGF21 and GDF15 are observed in the plasma of cancer patients and linked to poor prognosis, indicating their potential roles in the UPR^mt^ and association with cancer [8-11].

In *C. elegans* nerve cells, the polyglutamine tract of a specific length (PolyQ40) is expressed on the mitochondrial outer membrane. PolyQ40 not only interacts with mitochondria in nerve cells to affect mitochondrial membrane potential or protein input but also induces a mitochondrial stress response in distal tissues [12]. Additionally, unc-31 and serotonin mediate neuronal secretion to trigger the cell non-autonomous UPR^mt^ [12]. G protein-coupled receptor (GPCR) signaling also induces systemic UPR^mt^, and different forms of neuronal mitochondrial stress may cause systemic UPR^mt^ through different GPCR signaling pathways. Expression of SRZ-75 (a G-protein-coupled receptor) in chemical sensory ADL (amphid neurons with dual ciliated sensory endings) neurons causes non-autonomous UPR^mt^ in intestinal cells through the activation of GPCR-Gαq signaling and its downstream factor unc-73 [13]. However, the expression of SRZ-75 in the intestine does not induce the UPR^mt^, so neurons may play a complex role in coordinating the UPR^mt^ from different tissues.

**References**

1. Burtscher J, Soltany A, Visavadiya NP, Burtscher M, Millet GP, Khoramipour K, et al. Mitochondrial stress and mitokines in aging. Aging Cell. 2023;22(2):e13770.

2. Durieux J, Wolff S, Dillin A. The cell-non-autonomous nature of electron transport chain-mediated longevity. Cell. 2011;144(1):79-91.

3. Kim KH, Jeong YT, Oh H, Kim SH, Cho JM, Kim YN, et al. Autophagy deficiency leads to protection from obesity and insulin resistance by inducing Fgf21 as a mitokine. Nat Med. 2013;19(1):83-92.

4. Jena J, García-Peña LM, Pereira RO. The roles of FGF21 and GDF15 in mediating the mitochondrial integrated stress response. Front Endocrinol (Lausanne). 2023;14:1264530.

5. Lu HJ, Koju N, Sheng R. Mammalian integrated stress responses in stressed organelles and their functions. Acta Pharmacol Sin. 2024;45(6):1095-114.

6. Keipert S, Ost M. Stress-induced FGF21 and GDF15 in obesity and obesity resistance. Trends Endocrinol Metab. 2021;32(11):904-15.

7. Salminen A, Kaarniranta K, Kauppinen A. Regulation of longevity by FGF21: Interaction between energy metabolism and stress responses. Ageing Res Rev. 2017;37:79-93.

8. Lu W, Li X, Luo Y. FGF21 in obesity and cancer: New insights. Cancer Lett. 2021;499:5-13.

9. Peñas A, Fernández-De la Torre M, Laine-Menéndez S, Lora D, Illescas M, García-Bartolomé A, et al. Plasma Gelsolin reinforces the diagnostic value of FGF-21 and GDF-15 for mitochondrial disorders. Int J Mol Sci. 2021;22(12).

10. Spanopoulou A, Gkretsi V. Growth differentiation factor 15 (GDF15) in cancer cell metastasis: from the cells to the patients. Clin Exp Metastasis. 2020;37(4):451-64.

11. Sui Y, Chen J. Hepatic FGF21: Its emerging role in inter-organ crosstalk and cancers. Int J Biol Sci. 2022;18(15):5928-42.

12. Berendzen KM, Durieux J, Shao LW, Tian Y, Kim HE, Wolff S, et al. Neuroendocrine coordination of mitochondrial stress signaling and proteostasis. Cell. 2016;166(6):1553-63.e10.

13. Liu Y, Zhou J, Zhang N, Wu X, Zhang Q, Zhang W, et al. Two sensory neurons coordinate the systemic mitochondrial stress response via GPCR signaling in *C. elegans*. Dev Cell. 2022;57(21):2469-82.e5.
