# Supplementary material for: A bird’s eye view of mitochondrial unfolded protein response in cancer: mechanisms, progression and further applications
Source: Cell Death Dis. 2024 Sep 11;15(9):667. doi: 10.1038/s41419-024-07049-y (PMC11390889; doi:10.1038/s41419-024-07049-y)
Supplement: Supplementary file 3 [file 41419_2024_7049_MOESM3_ESM.doc]

**Supplementary File 3. The relationship between SIRT3 and UPR^mt^ and oncogenesis.**

**SIRT3 and UPR^mt^**

SIRT3 is a mitochondrial deacetylase that plays a crucial role in maintaining mitochondrial health and function. SIRT3 influences various processes including energy balance, redox state, mitochondrial biogenesis, mitophagy and UPR^mt^ by modulating the acetylation status of proteins [1]. SIRT3 is induced by the oxidative stress [2]. SIRT3 deacetylates FOXO3α, and FOXO3α then enters the nucleus to promote the transcription of superoxide dismutase 2 (SOD2) and catalase, thereby reducing ROS levels and alleviating proteotoxic stress (Fig. 1) [3]. Mice with liver-specific knockout of SIRT3 showed hyperacetylation and disrupted mitochondrial protein metabolism [4]. A recent study suggests that the SIRT3/SOD2 signaling pathway alleviates myocardial ischemia-reperfusion injury (IRI) by inhibiting oxidative stress and apoptosis [5]. Moreover, drugs such as Qishen granules restrain oxidative damage and protect mitochondrial function through the SIRT3/Ac-SOD2 pathway [6]. Furthermore, while SIRT3 decreases with age in women, the role of ERα-involved UPR^mt^ pathways will become increasingly important, suggesting that the different UPR^mt^ pathways are complementary [7].

**The roles of SIRT3 in oncogenesis**

SIRT3 plays a crucial role in regulating cellular metabolism, oxidative stress, and apoptosis. Given its role in these cellular processes, SIRT3 has emerged as a potential therapeutic target in cancer research. In cells with excess ROS levels, UPR^mt^ activation may induce SIRT3 expression to reduce ROS to a level sufficient to be used as proliferative signaling molecules in cancer cells [8]. SIRT3 promotes the growth, metastasis and invasion of cancer cells [9, 10]. SIRT3 impacts the UPR^mt^ by binding to SOD2, which has a similar function to SOD1. Upregulation of SOD1 and activation of the UPR^mt^ can protect cells from mitochondrial damage and cell death. SOD1 plays a key role in mitochondrial communication through its accumulation in internal cell membranes and activates the UPR^mt^ by enhancing the ERα transcriptional activity [11]. The SIRT3-mediated UPR^mt^ may prevent the clearance of harmful mutated mtDNA and promote cancer cell metastasis. Consistently, mtDNA landscape studies have shown that the SIRT3/FOXO3α/SOD2 pathway activates the UPR^mt^ to enhance mitochondrial integrity and fitness to promote metastasis [12]. In fact, SIRT3 has a context-dependent role in cancer, acting as both a tumor promoter and a tumor suppressor [13-15]. For example, SIRT3 deacetylates isocitrate dehydrogenase (IDH2) to increase its activity, thus facilitating carcinogenesis [16]. IDH2 is an important enzyme catalyzing the oxidative decarboxylation of isocitrate to form α-ketoglutarate (α-KG) and NADPH. Hotspot mutations in IDH2 occur in a variety of cancers and are associated with cancer development [17]. Therefore, SIRT3 function may depend on the type of cancer, the cellular context, and the presence of various stressors or cell death triggers.

The development of SIRT3 inhibitors is of interest in cancer research because they have the potential to be used as anti-cancer therapies, especially for cancers that exhibit high SIRT3 activity or rely on specific SIRT3-regulated metabolic pathways [14, 18]. However, the context-dependent role of SIRT3 in cancer necessitates careful consideration in the development of targeted therapies.

**References**

1. Shen H, Ma W, Hu Y, Liu Y, Song Y, Fu L, et al. Mitochondrial sirtuins in cancer: A revisited review from molecular mechanisms to therapeutic strategies. Theranostics. 2024;14(7):2993-3013.

2. Papa L, Germain D. SirT3 regulates the mitochondrial unfolded protein response. Mol Cell Biol. 2014;34(4):699-710.

3. Inigo JR, Chandra D. The mitochondrial unfolded protein response (UPR(mt)): shielding against toxicity to mitochondria in cancer. J Hematol Oncol. 2022;15(1):98.

4. Cho CS, Lombard DB, Lee JH. SIRT3 as a regulator of hepatic autophagy. Hepatology. 2017;66(3):700-2.

5. Wu L, Yan X, Sun R, Ma Y, Yao W, Gao B, et al. Sirt3 restricts tumor initiation via promoting LONP1 deacetylation and K63 ubiquitination. J Transl Med. 2023;21(1):81.

6. Zhang J, Li W, Xue S, Gao P, Wang H, Chen H, et al. Qishen granule attenuates doxorubicin-induced cardiotoxicity by protecting mitochondrial function and reducing oxidative stress through regulation of Sirtuin3. J Ethnopharmacol. 2024;319(Pt 1):117134.

7. Jenkins EC, Chattopadhyay M, Germain D. Are the estrogen receptor and SIRT3 axes of the mitochondrial UPR key regulators of breast cancer sub-type determination according to age? Aging Cancer. 2021;2(3):75-81.

8. Prasad S, Gupta SC, Tyagi AK. Reactive oxygen species (ROS) and cancer: Role of antioxidative nutraceuticals. Cancer Lett. 2017;387:95-105.

9. Papa L, Hahn M, Marsh EL, Evans BS, Germain D. SOD2 to SOD1 switch in breast cancer. J Biol Chem. 2014;289(9):5412-6.

10. Papa L, Manfredi G, Germain D. SOD1, an unexpected novel target for cancer therapy. Genes Cancer. 2014;5(1-2):15-21.

11. Gao J, Feng Z, Wang X, Zeng M, Liu J, Han S, et al. SIRT3/SOD2 maintains osteoblast differentiation and bone formation by regulating mitochondrial stress. Cell Death Differ. 2018;25(2):229-40.

12. Kenny TC, Hart P, Ragazzi M, Sersinghe M, Chipuk J, Sagar MAK, et al. Selected mitochondrial DNA landscapes activate the SIRT3 axis of the UPR(mt) to promote metastasis. Oncogene. 2017;36(31):4393-404.

13. Ouyang S, Zhang Q, Lou L, Zhu K, Li Z, Liu P, et al. The double-edged sword of SIRT3 in cancer and its therapeutic applications. Front Pharmacol. 2022;13:871560.

14. Zhang J, Xiang H, Liu J, Chen Y, He RR, Liu B. Mitochondrial Sirtuin 3: New emerging biological function and therapeutic target. Theranostics. 2020;10(18):8315-42.

15. Zhang J, Ye J, Zhu S, Han B, Liu B. Context-dependent role of SIRT3 in cancer. Trends Pharmacol Sci. 2024;45(2):173-90.

16. Bergaggio E, Riganti C, Garaffo G, Vitale N, Mereu E, Bandini C, et al. IDH2 inhibition enhances proteasome inhibitor responsiveness in hematological malignancies. Blood. 2019;133(2):156-67.

17. Tommasini-Ghelfi S, Murnan K, Kouri FM, Mahajan AS, May JL, Stegh AH. Cancer-associated mutation and beyond: The emerging biology of isocitrate dehydrogenases in human disease. Sci Adv. 2019;5(5):eaaw4543.

18. Chen Y, Fu LL, Wen X, Wang XY, Liu J, Cheng Y, et al. Sirtuin-3 (SIRT3), a therapeutic target with oncogenic and tumor-suppressive function in cancer. Cell Death Dis. 2014;5(2):e1047.
